# Percussion Drilling Hole in Cu, Al, Ti and Ni Alloys Using Ultra-Short Pulsed Laser Ablation

**DOI:** 10.3390/ma13010031

**Published:** 2019-12-19

**Authors:** Wanqin Zhao, Haodong Liu, Xiaowei Shen, Lingzhi Wang, Xuesong Mei

**Affiliations:** 1 School of Materials Engineering, Shanghai University of Engineering Science, Shanghai 201620, China; M050119308@sues.edu.cn (H.L.); M050119311@sues.edu.cn (X.S.); M050318101@sues.edu.cn (L.W.); 2 State Key Laboratory for Manufacturing Systems Engineering, Xi’an Jiaotong University, Xi’an 710049, China; xsmei@xjtu.edu.cn; 3 Shanghai Collaborative Innovation Center of Laser Advanced Manufacturing Technology, Shanghai 201620, China

**Keywords:** micro-deep hole, percussion drilling, metals, ultrashort pulsed laser

## Abstract

Comparative study on micro-deep hole drilling by picosecond ultra-short pulse laser in four metals, including copper, aluminum alloy, titanium alloy, and nickel alloy, was presented. Destructive testing was performed to measure the depth, shape, and side-wall morphology of micro-deep holes in this study. Diameters and depths of micro-deep holes in four metals ablated using the same processing parameters were compared. The multiple thresholds of metals had been calculated. Relationships between hole dimensions and multiple thresholds (the average ablation threshold, the ablation threshold and the thermal threshold) and physical properties (for example, the heat capacity, the melting temperature, the optical penetration depth and the absorbance, etc.) of the four metals are examined. Furthermore, the surface and side-wall morphologies of the micro-deep holes in four metals were demonstrated. The influence mechanisms, especially the side-wall morphology, were discussed, such as the thresholds, conical emission, self-cleaning effect, physical properties of metals, the energy transmission, the plasma effect, thermochemical reaction, and so on.

## 1. Introduction

Ultrashort pulsed laser micro-hole ablation offers lots of advantages, including breaking through the diffraction limit so the low-micron even nanometer holes could be expected [1,2], processing holes with diverse aspect ratios through adjusting multiple parameters [3,4]. Besides, larger diameter and a variety of shapes holes, such as triangles, squares, diamonds, etc., have been ablated through trepanning and helical drilling with the relative movement between the laser and sample [5,6,7,8,9]. Meanwhile, a micro-deep hole produced by ultra-short laser pulsed drilling, also known as ablation, has been used to fabricate metal components for widespread applications such as nozzles for the aerospace industry [10], printing field [11], and through-holes in the photoelectronic field [12,13]. It has been paid much attention to ultrashort pulsed laser micro-hole ablation in metals.

A few studies on the side-wall morphology of micro-deep hole have been performed in metals, however. Two main issues are, first, holes cannot be detected the real-time during the ablation process because metals are not transparent; thus, measurements must be performed afterward. Second, the depth and side-wall morphology of micro-deep holes cannot be directly measured using available methods, such as laser scanning confocal microscopy (LSCM), atomic force microscopy (AFM), and stylus profilometry (SP). Effective measurement methods are destructive and require metal samples first to be ground and polished and subsequently imaged. In this way, the side profile and side-wall morphology of drilled holes have been obtained by scanning electron microscopy (SEM). For example, Li et al. [14] successfully used SEM to examine the depth and side-wall morphology of micro-deep holes in a nickel (Ni) alloy created by femtosecond laser drilling. Similarly, Vahid et al. [15] used a high-resolution X-ray tomography (XRT) to examine micro-deep holes in silicon nitride produced by ultrashort pulsed laser drilling. Now, only the side profile, and not the side-wall morphology, can be effectively analyzed in this way.

Furthermore, the comparative study on multiple metals drilling is less. For example, Campbell et al. reported hole ablation in aluminum, copper, stainless steel, and titanium, but only the hole depth was studied, and they were less than 25 µm [16]. By comparing the average drilling rate (nm/pulse) and energy efficiency (mm^3^/J), Spiro et al. investigated picosecond pulsed laser hole drilling with three different wavelengths in five metals: aluminum, nickel, stainless steel, molybdenum, and tungsten [17]. In another study, Ahmmed et al. introduced a surface texturing method using a femtosecond laser to induce micro to nanometer holes in titanium, stainless steel, aluminum, and copper [18].

Overall, ultrashort pulsed laser micro-hole ablation is generally concerned about the dimension and the surface morphology around the hole periphery, but less about the side-wall morphology of micro-deep hole based on the limitation of the detection. However, the side-wall morphology of the micro-deep hole in metals ablated by ultrashort pulsed laser is very important. For example, the difference in the side-wall morphology directly leads to the difference in the laser effect, which further affects the distribution state of energy, that is, the state of material removal. For the practical application, the side-wall morphology of hole determines the anti-corrosion characteristics, adhesion characteristics, and the crack generation mechanism, etc. In this paper, a comparative study of micro-deep holes fabricated by ultra-short pulsed laser percussion drilling in four metals, including Cu, Al alloy, Ti alloy, and an Ni alloy, is presented. Multiple thresholds were calculated for the metals, and the relationships between hole dimensions and the thresholds were determined. In addition, the physical properties of the metals are discussed. The surface and side-wall morphologies of micro-deep holes in the four different metals are compared.

## 2. Experimental Setup and Procedure

**Figure 1:**
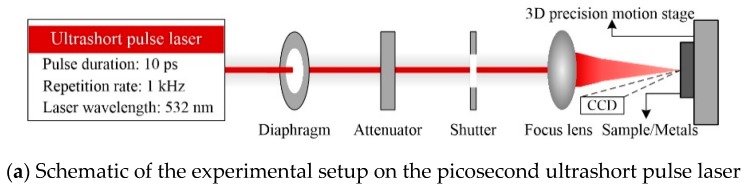

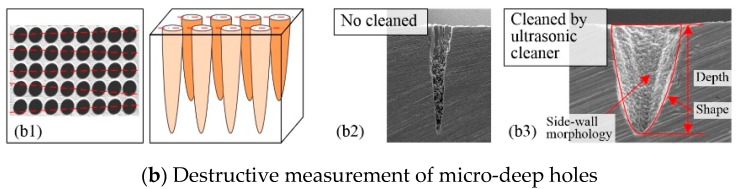
Schematic illustration of the experimental setup and the destructive measurement of the hole.

The laser source used in the experiments was a neodymium-vanadate (IC-1500 ps Nd:VAN REG AMP, High Q Laser, Rankweil, Austria) laser with a pulse duration of 10 ps. The laser wavelength was 532 nm, and the laser beam was linearly polarized with a repetition rate of 1 kHz. A laser beam with a radius of about 19 µm was directly focused on the surface of samples, including Cu (purity 99.9 wt.%), Al alloy (1050; purity 99.5 wt.%), Ti alloy (Ti6Al4V), and Ni alloy (Inconel 718), and focused using an F-theta lens with a focal distance of 150 mm. In addition, the maximum laser power for a wavelength of 532 nm was approximately 135 mW. Accordingly, the maximum laser energy was 135 µJ when combined with a repetition rate of 1 kHz, and the laser energy had been adjusted from 2 µJ to 135 µJ by the attenuator. Samples were placed on a 3D motorized translation stage (Owis, Staufen Im Breisgau, Germany), allowing accurate high-precision control of the laser position. Moreover, an accurate imaging system with a CCD (charge-coupled device) camera was used, enabling high-resolution visualization of the ablation zone on sample surfaces. A schematic illustration of the experimental setup with the ultra-short pulsed laser is shown in Figure 1a.

Experiments were performed under ambient conditions. Afterward, the surface morphologies of the holes were observed by SEM (Hitachi, Tokyo, Japan), and the diameters of the holes were measured on the surface of SEM micrographs. The single hole diameter was obtained by averaging the maximum and minimum values. At least five holes were measured for the same processing parameters, and then the average was figured up. Depths of the shallow holes were measured by LSCM (Olympus, Tokyo, Japan). Destructive testing was used for holes with depths outside the LSCM measurement range, as shown in Figure 1b. Briefly, multiple holes were drilled on the surface of samples, as shown in Figure 1b1, then the laser-ablated holes were ground and polished using sandpapers and polishing agents, followed by cleaned in an ultrasonic bath containing an acetone cleaner, as illustrated in Figure 1b2,b3. Holes were then imaged by SEM and hole depth, shape, and side-wall morphology was obtained from SEM micrographs of each sample, as shown in Figure 1b3. In addition, energy dispersive spectrometry (EDX) was performed to compare the chemical composition of the metals after drilling. One more, ultraviolet and visible light spectrophotometry (UVLS, Shimadzu, Kyoto, Japan) was used to measure the reflectivity of the metals, and the corresponding absorptivity was calculated.

## 3. Results and Discussion

### 3.1. Hole Dimensions in Different Metals

**Figure 2:**
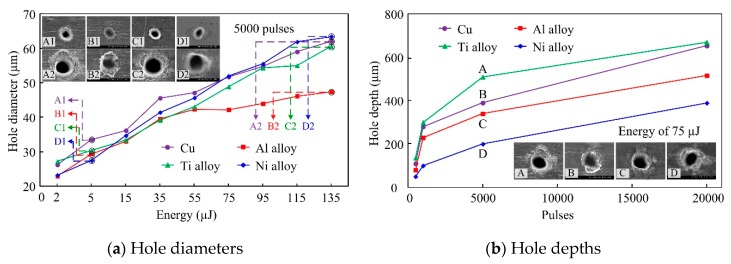
Dimensions of drilled holes in the four different metals.

Differences in hole dimensions in the four metals are illustrated in Figure 2, hole diameters varied with the laser energy. When laser energy of 2 µJ was used, hole diameters were similar for the Al alloy and Ni alloy and for the Cu and Ti alloy. Diameters of the Cu and Ti alloy were larger than those of Al alloy and Ni alloy, as shown in Figure 2a. As the laser energy increased while remaining below 55 µJ, very little difference was observed between hole diameters in the Al alloy and Ti alloy. However, when the energy was increased above 55 µJ, hole diameters in the Ti alloy gradually became larger than those in the Al alloy. When the laser energy was less than 75 µJ, hole diameters in Cu were larger than those in the Ni alloy; whereas, the opposite trend was observed once the laser energy increased above 75 µJ. Finally, hole diameters in the order of increasing size were Ni alloy, Cu, Ti alloy, and Al alloy, as shown in Figure 2a. On the other hand, for the depth of the holes, as shown in Figure 2b, The deepest hole was observed in the Ti alloy, and then Cu, followed by the Al alloy, and finally, the Ni alloy.

Differences in the hole dimensions between the four metals illustrated in Figure 2, may be associated with the thresholds of ablated metals by the ultra-short pulsed laser. First, the laser energy is absorbed by the material and then converted into source energy as the laser and material interact. When the energy absorbed by the material exceeds the ablation threshold of the material, some of the material is removed, and the holes are gradually formed. Nevertheless, energy deposited inside the material is generally higher than the ablation threshold of the material, leading to strong heating effects. Therefore, another material threshold can also be defined, denoted the thermal threshold [19]. Furthermore, both the ablation threshold and thermal threshold can be calculated from the linear line of fit between the logarithm of the laser peak fluence versus the ablated hole diameter [20,21].

**Figure 3:**
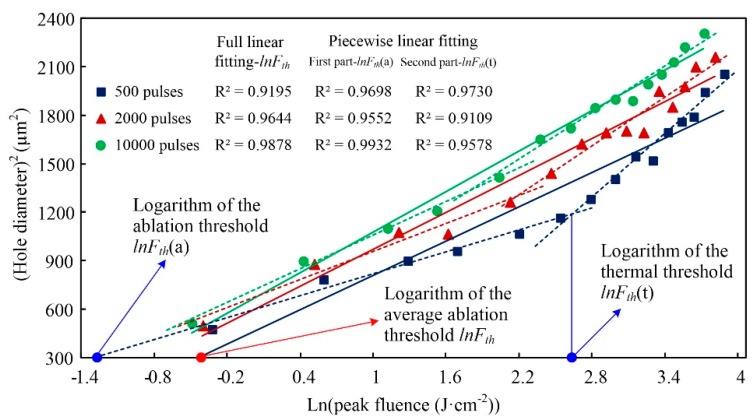
Linear line of fit of the logarithm of laser peak fluence versus a squared hole diameter in the Al alloy.

Herein, the ablation of the Al alloy is taken as an example, and Figure 3 shows the linear line of fit. Solid lines depict the linear line of fit for the total data and logarithms of the average ablation thresholds, denoted $lnF_{th}$, which intercept the horizontal axes. Dotted lines represent the piecewise linear line of fit, and where it intercepts the horizontal axis from the first parts are the logarithms of the ablation thresholds, denoted $lnF_{th}\left(a\right)$, and the logarithms of the thermal thresholds are the values on the horizontal axes being from the intersections between the first and the second parts dotted lines, denoted as $lnF_{th}\left(t\right)$. By calculating the inverse logarithm, the average ablation threshold $F_{th}$, the ablation threshold $F_{th}\left(a\right)$, and the thermal threshold $F_{th}\left(t\right)$ were obtained for multiple pulse groups. Finally, the single-pulse threshold was determined according to the empirical incubation model [22].

**Figure 4:**
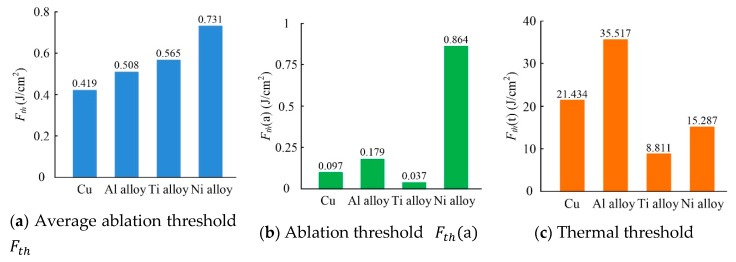
Single-pulse thresholds of the four different metals.

A comparison of the average ablation threshold $F_{th}$, ablation threshold $F_{th}\left(a\right)$, and thermal threshold $F_{th}\left(t\right)$ with single-pulse of the four metals are presented in Figure 4. Many differences can be observed between the material thresholds based on the linear fit for each of the different metals. For example, the Ni alloy was found to have the highest average ablation threshold and ablation threshold but exhibits only the third-highest thermal threshold of the four metals. Instead, the Al alloy has the highest thermal threshold, as shown in Figure 4.

No corresponding relationships were found between any of the thresholds and hole diameter. The opposite trend can be observed between the ablation threshold $F_{th}\left(a\right)$ and hole depth. In other words, as the ablation threshold $F_{th}\left(a\right)$ of a material reduces, hole depth increases. These results differ from previously published and widely accepted conclusions suggesting that a larger hole diameter indicates a smaller threshold value [23]. The same relationship between threshold and hole diameter was not observed in this study. In fact, numerous factors were found to determine the ablated hole diameter, not just threshold values. However, consistent with the findings of this study, the ablated hole depth is known to decrease as the ablation threshold increases.

Characteristics of metals, such as heat capacity, melting temperature, optical penetration depth, and absorptivity of the material, as shown in Table 1, are also likely to influence hole dimensions. Of the four metals, the heat capacity of the Ni alloy is highest, that is, the Ni alloy requires the most energy to increase its temperature. Combined with the higher melting temperature of the Ni -alloy, this means ablation is difficult in Ni alloy, and shallow holes were obtained. For the Al alloy, the smallest optical penetration depth was observed, resulting in energy only being absorbed in the shallow layers, thus limiting the depth of the holes produced. The deepest optical penetration depth was observed in Cu, and was around twice the depth of Al alloy; thus, holes in Cu are deeper than those formed in the Al alloy. Finally, the Ti alloy has the lowest heat capacity and the highest absorbance for the 532-nm laser wavelength used in the experiments; therefore, the deepest holes were formed in the Ti alloy. It should be noted that analyses comparing the thresholds, physical properties of metals, and hole depths were qualitative, and factors influencing the hole depth produced by ultra-short pulsed laser ablation are numerous and go beyond just the thresholds and physical parameters discussed in this paper.

### 3.2. Hole Morphology in Different Metals

#### 3.2.1. Surface Morphology

**Figure 5:**
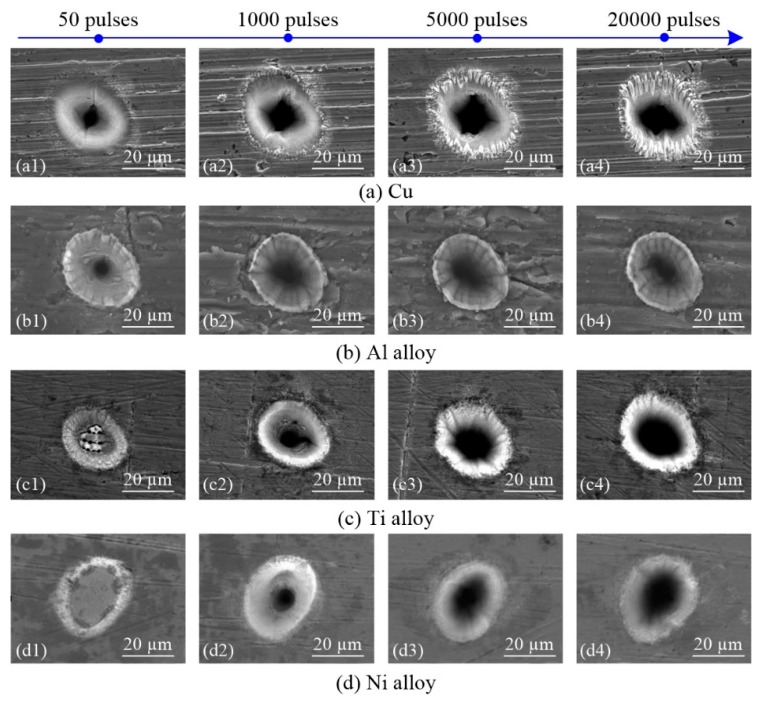
SEM images of holes in four different metals produced by ultrashort pulsed laser ablation (energy of 5 µJ).

Figure 5 presents SEM images of holes produced in four different metals by ultra-short pulsed laser ablation using laser energy of 5 µJ. In Cu, using 50 pulses resulted in very little molten metal or burrs on the hole surface, as shown in Figure 5a. However, when the number of pulses was increased to 1000, the molten metal and burrs increased. Continued to increase up to 5000 pulses, in addition, numerous short and deep ravine structures appeared along the direction of the hole depth. When the number of pulses was increased to 20,000, the ravine structures became longer and deeper. For the Al alloy, obvious molten material was observed around the hole from 50–20,000 pulses and the molten material was distributed pile up around the holes, as shown in Figure 5b, which is not similar to the results of Cu ablation. Holes that formed in Ti alloy were very shallow when 50 pulses were used, the bottom of the hole was composed of pinholes and coarse particles while the rest part of the hole was dominated by ripples. The hole depth increased as the number of pulses increased, and a small amount of molten material appeared around the hole, as shown in Figure 5c. In Ni alloy, extremely shallow holes were observed when 50 pulses are used, and the whole surface is composed of ripples. As the number of pulses increased, the hole depth also increased, and no molten material was observed around the holes, as shown in Figure 5d.

Comparing Figure 5a1–d1 with the same energy of 5 µJ and 50 pulses, ablation is more intense in Ti alloy than in Ni alloy based on the different hole morphologies obtained. A coarse particle topography was exhibited in Ti alloy, and pinholes were observed, whereas holes surface in Ni alloy exhibit a more rippled topography. At the same time, holes of a certain depth were drilled in Cu and Al alloy, showing that ablation in Cu and Al alloy are more intense than in Ti alloy and Ni alloy. In brief, Ni alloy is the most difficult to machine by ultra-short pulsed laser ablation, followed by Ti alloy; however, it is impossible to determine the difficulty of the ablation of Al alloy and Cu. Furthermore, thresholds of the material can be used to assess processing difficulty. Materials with higher thresholds are typically more difficult to process. Figure 5a1–d1 shows that Ni alloy has the highest threshold, followed by Ti alloy, then Al alloy and Cu. These results are consistent with calculations based on the linear line of fit, that is, the average ablation threshold $F_{th}$, as shown in Figure 4.

**Figure 6:**
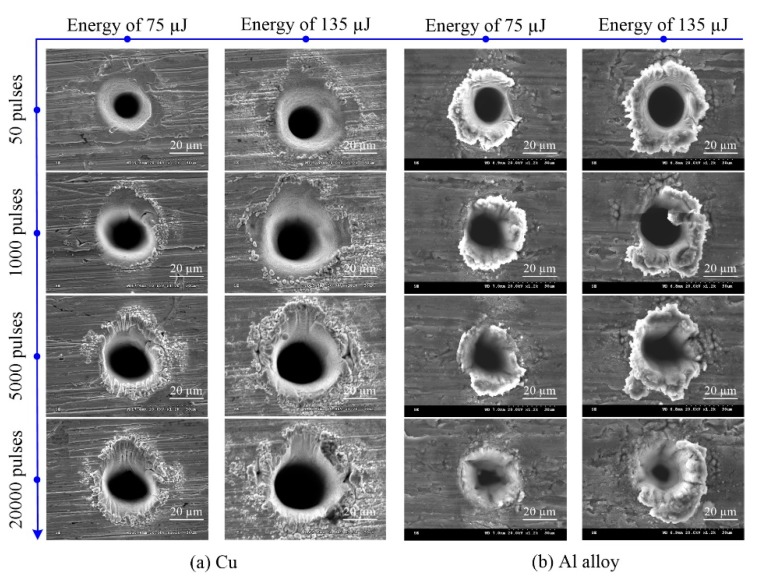

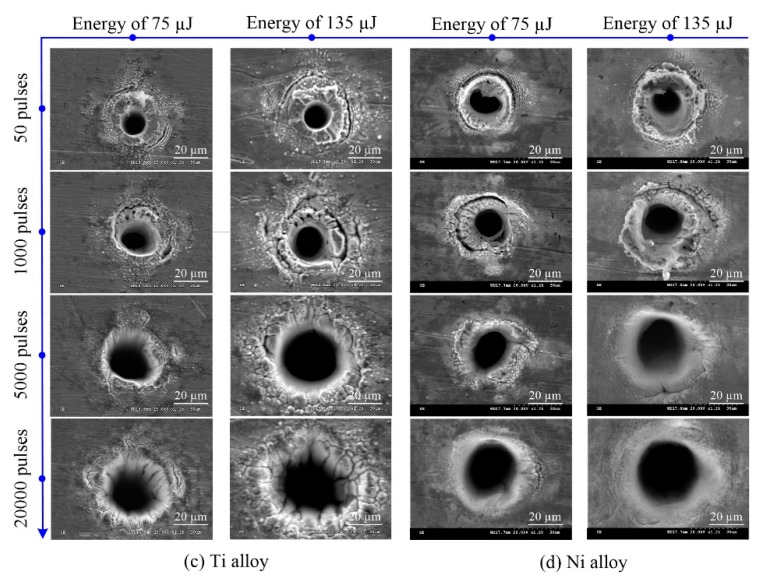
SEM images of holes in four different metals produced by ultra-short pulsed laser ablation.

Hole surface morphologies are shown in Figure 6 for increasing laser energy. In Cu, molten material was scattered around the hole, and the quantity and splash range significantly increased as the pulse energy and the number of pulses increased; moreover, short and deep ravine structures are produced, mainly observed in Figure 6a when the number of pulses is 5000 or 20,000. In Al alloy, molten material builds up around the hole, as shown in Figure 6b. Further, as the number of pulses increased at the same laser energy, molten material around the top surface of the hole decreased, and in some cases, disappeared. Between tens and thousands of pulses, for example 50 and 1000 pulses, shown in Figure 6c,d, resulted in irregularly shaped holes in Ti alloy and Ni alloy induced by conical emission (CE) [29]. The same structure was reported for other metals machined by ultrashort pulsed laser ablation, such as SS304 [30] and steel [31]. Under the same processing parameters, no ring structure appeared in Cu and Al alloy, suggesting that CE does not necessarily induce a ring structure but is still affected by material properties. Furthermore, as the number of laser pulses increased, the ring structure disappeared, and the amount of molten material decreased, or in some cases, even disappeared, as shown in Figure 6c,d.

Overall, self-cleaning effects were observed for Al alloy, Ti alloy, and Ni alloy during ultra-short laser pulsed ablation, suggesting that subsequent pulses remove molten material from around the hole surface, thereby obtaining micro-deep holes with high-quality surface morphologies [32]. Furthermore, optimal parameters required to achieve these self-cleaning effects depend on the type of metal, for example, the optimal parameters of Al alloy are energy of 75 µJ and 20,000 pulses, and of Ni-alloy, energy of 135 µJ and 20,000 pulses. However, no self-cleaning effects were observed in Cu in our experiments. The reason may be that unlike the other metals, molten material is distributed throughout the hole instead of building up around the top of the hole.

Physical properties also influence hole surface morphology, such as those presented in Table 1. A large amount of molten material accumulated around holes in the Al alloy, and this may be associated with its shorter electron-phonon coupling time, which governs how quickly heat travels through the material, even if the difference is only a few picoseconds, as shown in Table 1. The thermal conductivity of Al alloy is higher than other metals, resulting heat being more readily dissipated into the surrounding area. In addition, Al alloy has both a lower melting temperature and a smaller optical penetration depth. As a result, laser energy will be mainly absorbed by the surface, resulting in a buildup of molten material around the hole.

Compared to Al alloy, the higher thermal conductivity and melting temperature of Cu suggest that it will have a smaller thermal threshold, and the optical penetration depth of Cu is deeper, causing laser energy to deliver into the material easily. Therefore, less molten material was found around the ablated holes in Cu than in the Al alloy. Since Ni alloy has a higher thermal conductivity and lower melting temperature, its thermal threshold will be larger than the Ti alloy, leading to more molten material around the holes in Ni alloy. However, these observations only apply to ablation with fewer than several thousand pulses of Ti and Ni alloys since the hole surface morphology is determined by the self-cleaning effect when the number of pulses is high.

#### 3.2.2. Side-Wall Morphology

**Figure 7:**
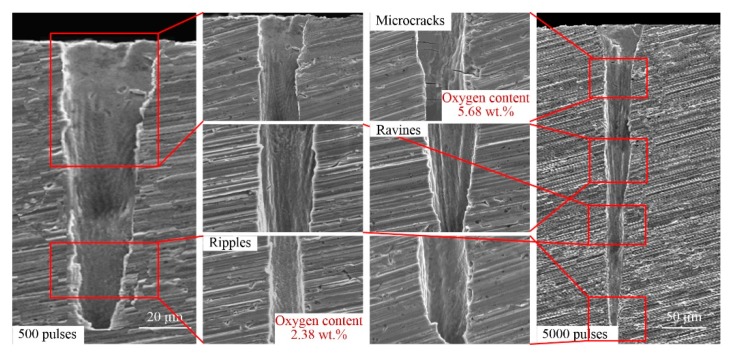
Representative SEM images of hole side-wall morphology in Cu produced by ultrashort pulsed laser ablation (energy of 75 µJ).

**Figure 8:**
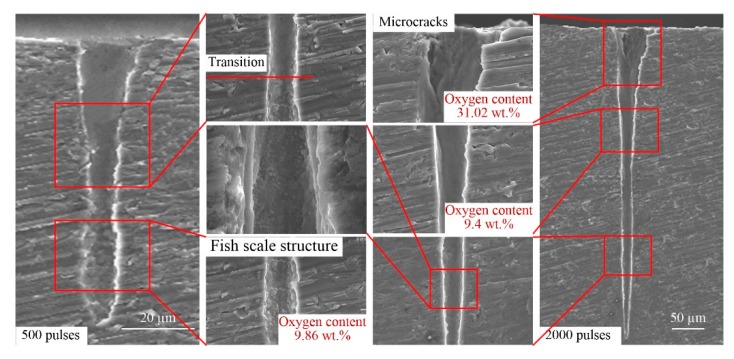
Representative SEM images of hole side-wall morphology in Al alloy produced by ultrashort pulsed laser ablation (energy of 75 µJ).

**Figure 9:**
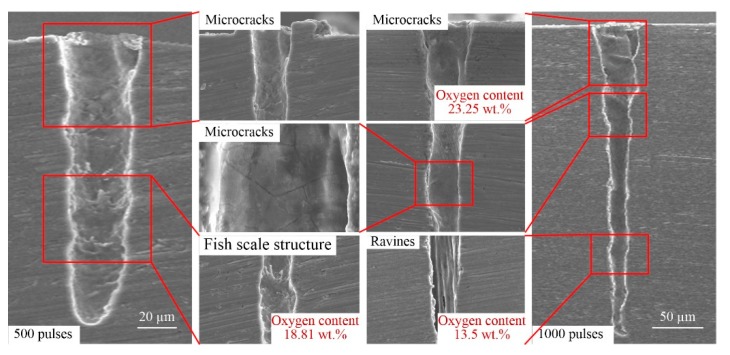
Representative SEM images of hole side-wall morphology in Ti alloy produced by ultrashort pulsed laser ablation (energy of 75 μJ).

**Figure 10:**
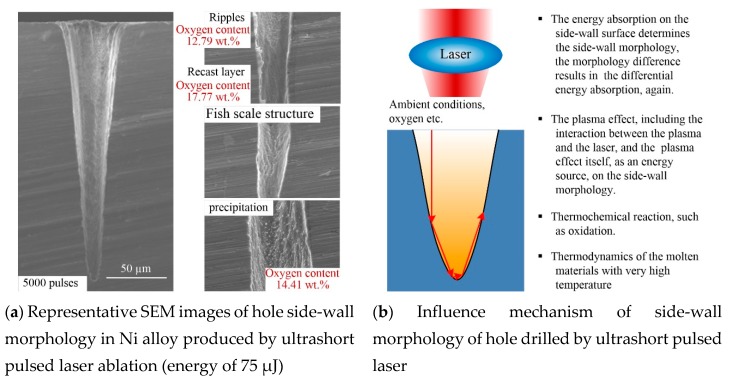
Representative SEM images of hole side-wall morphology in the Ni alloy and the influence mechanism of the hole side-wall morphology.

At present, the only method for measuring the side-wall morphology of micro-deep holes is first grinding and polishing samples and then performing SEM, as shown in Figure 1b. Figure 7, Figure 8, Figure 9 and Figure 10 show representative SEM images of the side profile of holes formed in the four metals by ultra-short pulsed laser ablation with an energy of 75 µJ. All holes are cone-shaped, wider at the top and narrower at the bottom, and ablated holes formed with more pulses have a larger taper than those produced by fewer pulses, with the exception of holes ablated with 500 pulses in Al alloy. As shown in Figure 8, the narrowest transverse hole diameter is located in the middle part of the hole.

Side-wall surface morphology appears smooth near the inlet, but as the hole depth increases, side-wall surfaces of holes become rougher. Ripples, microcracks, ravines, precipitation, recast layers, and a fish-scale structure are characteristic of the side-wall surface. For example, the fish-scale structure was observed on the lower side-wall surface in Al alloy, Ti alloy, and Ni alloy, as shown in Figure 8, Figure 9 and Figure 10, as well as distinct precipitation in Ni alloy (Figure 10). Additionally, EDX (energy dispersive spectrometry) measurements show that the oxygen content was lower around the fish-scale structure than in the recast layer, and higher near ripples, suggesting that fish-scale structures occur due to melting and evaporation processes [33]. Moreover, fish-scale structures can be clearly observed on hole surfaces formed by laser ablation with 500 pulses in Al alloy and Ti alloy. In particular, a clear transition on the side-wall of the hole in Al alloy can be observed, as shown in Figure 8.

Microcracks worsen the mechanical properties of metal; however, this varies for different metals. Microcracks that formed in the laser-ablated Cu were perpendicular to the direction of the hole depth, and they are width and depth shown in Figure 7. In comparison, microcracks in Al alloy and Ti alloy were similar, and all of the microcracks were short and narrow with a non-uniform distribution, as shown in Figure 8 and Figure 9. It should be noted that microcracks typically occur on the surface of recast layers, which is where the highest oxygen content can be found compared to other morphological features. Taking ablation in the Al alloy as an example, the oxygen content was 31.02 wt.%, 9.4 wt.%, and zero on the recast layer, rippled area, and substrate, respectively. Therefore, it can be concluded that brittleness of the material increases due to re-solidification and adhesion of molten metal onto the side-walls of the hole, and microcracks formed in the recast layer, owing to the high oxygen content and high thermal stress. Thus, avoiding oxidation by performing ultra-short pulse laser processing in vacuum or inert gases may be an effective way of preventing microcracks.

For the formation mechanism of the side-wall morphology of hole drilled by an ultrashort pulsed laser, it mainly contains four aspects, as shown in Figure 10b. First, the laser energy is absorbed by the material, and the material is removed with various states, such as liquid, vapor, and plasma, etc. At this point, the different energy absorbed by different positions of the hole side-wall leads to the difference in the side-wall morphology of the hole. Furthermore, the morphology difference results in differential energy absorption. Second, the plasma effect.

High-intensity laser would ionize air and material particles to form atmospheric plasmas and material plasmas [34]. Plasmas, either on the surface of the hole or inside the hole, would interact with the laser, which would affect the laser beam propagation and its energy state, and then affect the side-wall morphology of the hole. In addition, the plasma is also an energy source and influence the side-wall morphology. Third, the high energy field during laser processing would induce the thermochemical effect, such as oxidation. It could be seen from Figure 7, Figure 8, Figure 9 and Figure 10 that the oxygen content on the side-wall surface was significantly higher than the one of the substrate. At last, there are some effects of thermodynamics of the molten materials with very high temperatures on the side-wall morphology.

## 4. Conclusions

This paper presented a comparative study on micro-deep holes drilling by picosecond ultra-short pulse laser in four metals: Cu, Al alloy, Ti alloy, and Ni alloy. Different holes diameters were obtained in the metals as the laser energy was increased. The deepest hole was observed in the Ti alloy, followed by Cu, then Al alloy, and finally, Ni alloy. The average ablation thresholds, ablation thresholds, and thermal thresholds of four metals had been calculated. The influence of multiple thresholds and physical characteristics of four metals on the dimensions of ablated holes were discussed. In the experiments, it has been found that there was the opposite trend between the ablation thresholds and hole depths. When the heat capacity and melting temperature of metal were high, or the optical penetration depth was small, the ablated holes were shallow. When there were the low heat capacity and the high absorbance, the holes were deep.

Each metal exhibited unique morphological characteristics. For example, holes surfaces in Cu have short, and deep ravine structures along the direction of the holes depth, and molten material are distributed throughout the hole. In contrast, molten material builds up around the holes surface in Al alloy during ultra-short pulsed laser ablation, the self-cleaning properties of Al, Ti, and Ni alloys resulted in micro-deep holes with high-quality surfaces. Moreover, all of the holes were cone-shaped, and the characteristic morphologies on the surfaces of the side walls contained ripples, microcracks, ravines, fish-scale structures, precipitation, and recast layers. Some differences in morphology were also observed between the different metals, such as the direction and scale of microcracks. Furthermore, possible mechanisms responsible for the differences among metals after ablation were discussed, such as the multiple thresholds, conical emission, self-cleaning effect, physical properties of metals, and so on. Besides, the influence mechanism of hole side-wall morphology also had been demonstrated.

## Figures and Tables

**Table 1:** Physical properties of the four metals [24,25,26,27,28].

	Cu	Al Alloy	Ti Alloy	Ni Alloy
Heat capacity *C_l_* (× 10^6^ J m^−3^ K^−1^)	3.46	2.43	2.33	4.1
Melting temperature *T_m_* (K)	1337	933	1941	1609
Optical penetration depth λ (nm)	13.5	7.5	/	14.5
Thermal conductivity *K* (W m−3 K−1)	401	238	7.2	11.4
Electron-phonon coupling constant *g* (× 10^16^ W m^−3^ K^−1^)	10	56.9	40	36
Electron-phonon coupling time $\tau_{ep}$ = *C_l_* (g/ps)	34.6	4.3	5.8	11.4
Absorptivity at 532 nm wavelength, tested (%)	61.41	48.7	64.71	65.48

Parts of data are referred from the elements Al, Ni, and Ti.
